# Maternal Obesity and Excessive Gestational Weight Gain Influence Endocannabinoid Levels in Human Milk Across Breastfeeding: Potential Implications for Offspring Development

**DOI:** 10.3390/nu17081344

**Published:** 2025-04-14

**Authors:** Tatiana F. Pontes, Gabriel Reis, Gustavo R. C. Santos, Henrique M. G. Pereira, Gilberto Kac, Ana L. L. Ferreira, Isis H. Trevenzoli

**Affiliations:** 1 Laboratório de Endocrinologia Molecular, Instituto de Biofísica Carlos Chagas Filho, Universidade Federal do Rio de Janeiro, Rio de Janeiro 21941-971, Brazil; pontestf@gmail.com; 2 Laboratório Brasileiro de Controle de Dopagem, Instituto de Química, Universidade Federal do Rio de Janeiro, Rio de Janeiro 21941-853, Brazil; 3 Observatório de Epidemiologia Nutricional, Instituto de Nutrição Josué de Castro, Universidade Federal do Rio de Janeiro, Rio de Janeiro 21941-853, Brazil; gilberto.kac@gmail.com (G.K.); analorenaferreiira@gmail.com (A.L.L.F.)

**Keywords:** obesity, breast milk, endocannabinoids, human milk composition, leptin, insulin

## Abstract

**Background/Objectives:** Endocannabinoids are endogenous bioactive lipids that promote neurodevelopment and positive energy balance. Increased levels of endocannabinoids are associated with obesity, but the effect of maternal obesity on breast milk endocannabinoids across lactation is mostly unknown. **Methods:** Women from Rio de Janeiro (Brazil) (n = 92) were followed from the third trimester of pregnancy to 119 days postpartum, and milk samples were analyzed in the postpartum days 2–8 (T1), 28–47 (T2), and 88–119 (T3). We assessed the endocannabinoids anandamide (AEA) and 2-arachidonoylglycerol (2-AG) by high-performance liquid chromatography–mass spectrometry, leptin and insulin by immunoassay, and macronutrients by colorimetric assays in milk samples. **Results:** Milk AEA concentration was higher in T2 compared with T1 or T3, while 2-AG levels were higher in T2 and T3 compared with T1. Milk endocannabinoids were directly correlated with pre-pregnancy body mass index (BMI), gestational weight gain (GWG), and milk triglycerides. Triglyceride and leptin levels were higher in mature milk (T2 and T3) of women with BMI > 25 or excessive GWG. Adjusted linear regression models showed a positive association between excessive GWG and milk 2-AG (β = 1629; 95% CI: 467–2792; *p* = 0.008). **Conclusions:** The endocannabinoid levels are higher in mature milk from women with obesity or excessive GWG, which may impact offspring development and metabolism.

## 1. Introduction

Obesity or overweight is highly prevalent among women of reproductive age, reaching over 15% worldwide (World Health Organization, WHO, 2016). In the United States, the prevalence of pre-pregnancy obesity reaches nearly 30% of women [1,2], and in Brazil, it is 32.2%, according to the Brazilian National Survey on Child Nutrition (ENANI, 2019) [3]. Maternal obesity is an obstetric concern, increasing the risk for gestational diabetes, preeclampsia, preterm birth, and other complications for mothers and infants [4,5,6]. It has also been associated with changes in breast milk composition in humans [7,8,9] and animal models [10,11,12,13]. These nutritional and metabolic imbalances during critical periods of development may be associated with the developmental origins of health and disease (DOHaD), programming the long-term metabolic phenotype of the offspring [5,14]. The molecular mechanisms associated with the long-term impact of maternal obesity on offspring mainly involve epigenetic changes resulting in maladaptive gene expression and unfavorable metabolic phenotypes [15,16,17].

Obesity is commonly associated with an overactivation of the endocannabinoid system (ECS) in humans and animal models [18,19,20,21,22,23], but there are still controversies in the literature [24]. The ECS is a lipid signaling system comprising several endogenous cannabinoids (endocannabinoids), cannabinoid receptors, and metabolizing enzymes that synthesize or degrade these bioactive lipids. Anandamide (AEA) and 2-arachidonoylglycerol (2-AG) are the main endocannabinoids in mammals, which are synthesized from membrane phospholipids containing arachidonic acid (*n*-6 polyunsaturated fatty acid, *n*-6 PUFA). These major endocannabinoids present well-characterized effects mediated by the type 1 and type 2 cannabinoid receptors, including an increase in appetite and lipogenesis and a decrease in energy expenditure [19,25]. In early development, the ECS plays an important role in regulating neurogenesis, the proliferation of neural progenitors, cell lineage commitment, neuronal migration, axonal elongation, synaptogenesis, glial formation, postnatal myelination, and neural network formation [25,26,27,28]. Therefore, changes in the endocannabinoid levels and signaling in early life can potentially affect energy metabolism regulation and physiological brain development programming, which can have long-term adverse consequences.

During early development, breast milk is the optimal nutrition for neonates, providing water, nutrients, bioactive molecules, hormones, and microbiome, and, therefore, protecting infants while their immune system matures [29,30]. It was recently discovered that breast milk also contains endocannabinoids [31,32,33,34,35], but the effect of maternal nutritional status on milk endocannabinoid levels is mostly unclear. In the present study, we hypothesized that maternal obesity/overweight (BMI > 25) and excessive gestational weight gain (ex GWG) would be associated with an increased content of AEA and 2-AG in human breast milk. We further hypothesized that maternal dietary lipids would be correlated with the levels of endocannabinoids in breast milk because it has been demonstrated that *n*-3 PUFA decreases the AEA and 2-AG levels [36,37] and *n*-6 PUFA increases cannabinoid signaling [37,38] in other biological matrixes. Lastly, we hypothesized that there would be a differential profile of these endocannabinoids in breast milk across breastfeeding periods as babies’ needs change over time.

## 2. Materials and Methods

### 2.1. The Study Design and Eligibility Criteria

The data used in the present study were from a prospective cohort followed in a public health care center in Rio de Janeiro, Brazil. The participants’ recruitment lasted from February 2017 to April 2019. Details about the study design were previously described [8,39].

**Figure 1:**
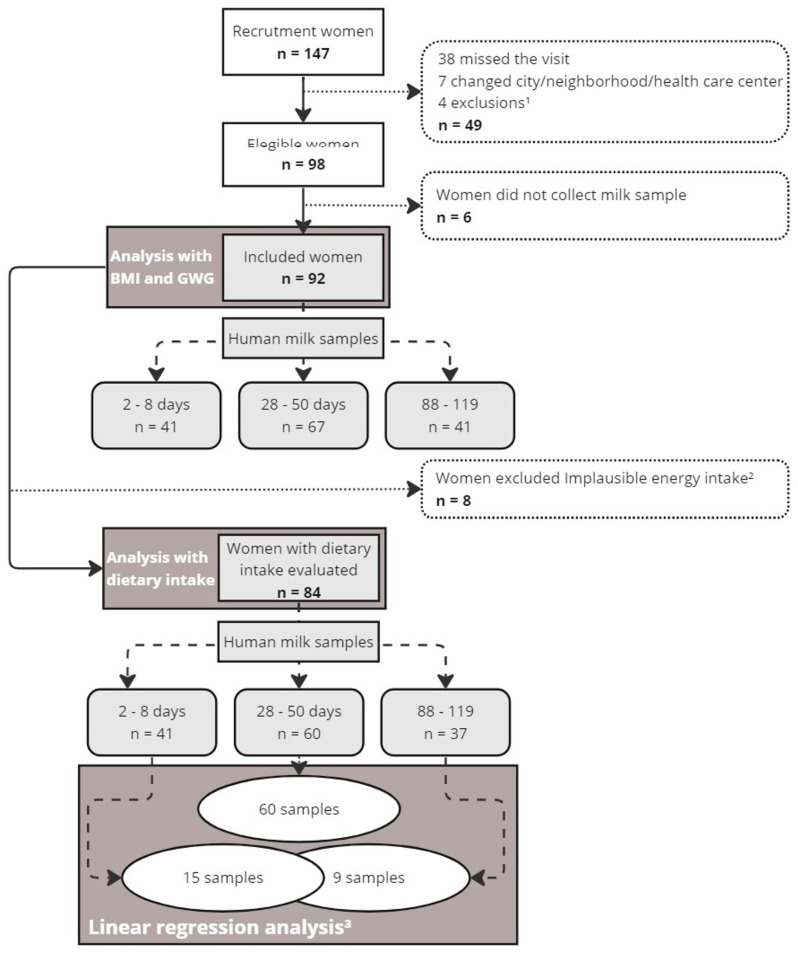
Study design. Flow diagram of the participants with anthropometric and dietary records and their milk samples collected for the analysis of endocannabinoids, hormones, proteins, and triglycerides during lactation in a women cohort of Rio de Janeiro, Brazil. BMI: body mass index; GWG: gestational weight gain. Note: ^1^ General exclusion included gestational diabetes, preeclampsia, preterm birth, and stillbirth. ^2^ Women with implausible energy intake include <600 or >6000 kcal/day. ^3^ In the linear regression analyses, all women with milk samples at 28–50 days postpartum (T2) (n = 60), women with samples at 2–8 days (T1) (n = 15, including those with samples also at the last visit), and those with samples exclusively at 88–119 days (T3) (n = 9) were included.

The current study analyzed data from a non-probabilistic sample of 92 women, with a baseline and three follow-ups, carried out at the following times, respectively: third trimester of pregnancy as baseline (between the 28th and 35th weeks of gestation) and postpartum days 2–8 (time 1—T1; sampling of colostrum), 28–50 (time 2—T2; sampling of 1-month milk), and 88 and 119 (time 3—T3; sampling of 3-month milk). Pregnant women who met the following eligibility criteria were included: age between 18 and 40 years old; time of pregnancy between the 28th and 35th weeks; singleton pregnancy and free of chronic non-transmissible diseases (diabetes, hypertension, heart diseases) and transmissible diseases; and singleton pregnancy. Participants were excluded when not breastfeeding during the follow-up (T1, T2, or T3) or when they developed gestational diabetes or preeclampsia, presented transmissible diseases, and delivered stillbirths or preterm infants (Figure 1).

Maternal pre-pregnancy BMI (weight (kg)/height (m)^2^) was calculated using self-reported pre-pregnancy weight (kg) and the height measured at 28–50 days postpartum (WHO 1995). Height was measured in duplicate using a stadiometer (Altura Exata, Belo Horizonte, Brazil). A third measurement was performed when a difference higher than 0.5 cm between the first two measurements was observed. In these cases, the mean of two similar measurements was used. The pre-pregnancy overweight/obesity variable was constructed based on the pre-pregnancy BMI categories (i.e., overweight/obesity BMI ≥ 25 kg/m^2^ and normal weight 18.5 < BMI < 25 kg/m^2^). Two women had a BMI < 18.5 kg/m^2^ (18.2 and 18.3 kg/m^2^) and were arbitrarily grouped in the normal weight category considering the cut-off proximity. Total gestational weight gain (GWG) was calculated as the difference between the body weight at the end of pregnancy and the self-reported pre-pregnancy weight. According to the Institute of Medicine (IOM, 2009) guidelines, GWG adequacy is categorized as follows: for women with a pre-pregnancy BMI < 18.5 kg/m^2^ (underweight), the recommended GWG is 12.5–18 kg; BMI between 18.5–24.9 kg/m^2^ (normal weight), it is 11.5–16 kg; BMI between 25–29.9 kg/m^2^ (overweight), it is 7–11.5 kg; and for those with a BMI ≥ 30 kg/m^2^ (obesity), it is 5–9 kg. GWG was considered excessive when above these recommended ranges [40]. 

A semiquantitative food frequency questionnaire (FFQ) was applied to estimate maternal dietary intake at the baseline time point. This instrument consisted of 85 food items and was previously validated in Brazil for estimating intakes of nutrients throughout gestation [41]. Estimates of dietary intake of total lipid, cholesterol, saturated, monounsaturated, and polyunsaturated lipids were considered. All dietary estimates were adjusted for total energy using the residual method [42].

The research protocol was carried out following the Declaration of Helsinki of 1975 and was approved by the Research Ethics Committees of the Municipal Secretary of Health and Civil Defense of the State of Rio de Janeiro (protocol number, CAAE: 49218115.0.3001.5279 approve on 8 July 2016) and by the research ethics committee of the *Maternidade Escola da Universidade Federal do Rio de Janeiro* (UFRJ) (CAAE: 49218115.0.0000.5275 approved on 25 February 2016).

### 2.2. Breast Milk Collection and Handling

The breast milk samples were collected at the public health care center during the T1, T2, and T3 interviews. This procedure was performed according to the Brazilian Network of Human Milk Banks protocol, regulated by the National Health Surveillance Agency (Brazil, 2007). The milk was collected manually, preferably by the participant, in the morning after breakfast.

Preceding breast milk extraction, general guidance on massage techniques to avoid engorgement and mastitis was provided. Subsequently, the nursing mother was instructed to wash her hands and use disposable head covers and face protection masks. The collections were performed directly in sterile, non-pyrogenic, ribonuclease (RNAse) and deoxyribonuclease (DNAse) free falcon tubes. In T1, 5 mL of milk was collected and later divided into 5 aliquots of 1 mL. At T2 and T3, 17 mL of milk were collected and distributed into 1 aliquot of 5 mL and 12 aliquots of 1 mL. Aliquoted samples were immediately frozen (−20 °C) and transported in a box with controlled temperature (1 °C to 5 °C) to be stored at −80 °C in the research laboratory for later analyses [7,39].

### 2.3. Milk Protein, Triglyceride, and Hormone Quantification

Protein breast milk concentration was assessed in skimmed milk by using the BCA PierceTM kit (Thermo Scientific, Waltham, MA, USA), and triglyceride concentration was determined in the whole milk by a colorimetric commercial kit (Bioclin, Quibasa Quimica, Belo Horizonte, Brazil), following the manufacturer’s recommendation. Leptin and insulin concentrations were assessed in skimmed milk by Milliplex kit (HMHEMAG-34K-07, Merck-Millipore, Burlington, MA, USA), following the manufacturer’s recommendations.

### 2.4. Milk Endocannabinoid Quantification

AEA and 2-AG milk concentrations were assessed by liquid chromatography–mass spectrometry (LC/MS) based on the method previously described for human milk [32] and rat milk [13] with slight adaptations. The assay was conducted using 180 μL of whole human milk or 180 μL of each standard of the calibration curve. The standard curve was obtained by serial dilution of the AEA and 2-AG standards (Cayman Chemical, Ann Arbor, MI, USA) in a 10% milk powder solution with the range of 0.03–5.0 ng/mL for AEA and 23–3000 ng/mL for 2-AG. The deuterated standards AEAd4 (1 ng) and 2-AGd5 (10 ng) (Cayman Chemical, USA) were added to each sample or curve as internal calibrators (20 μL). An ice-cold mixture of acetonitrile and PBS (1:1) was added to the samples and standards (1000 μL) for protein precipitation and lipid extraction. The proteins were separated by centrifugation (14,000× *g*, 4 °C, 5 min). The supernatant was acidified with 5 volumes of 5% phosphoric acid. Solid-phase extraction was performed using reversed-phase chromatography cartridges OASIS HLB (Waters Corp., Milford, MA, USA) following the manufacturer’s instructions. The lipid fraction was eluted from the columns with 1 mL of acetonitrile at room temperature. The eluate was dried under nitrogen steam at 32 °C for 20 min and resuspended in 100 μL of methanol for endocannabinoid detection (in triplicate) by LC-MS.

The chromatographic separation was performed in a reversed-phase column ACE CORE-25A-0502U (2.5 μm, 50 mm × 2.1 mm) at 40 °C. The mobile phases comprised (A) H2O with 5 mM ammonium formate and 0.1% formic acid and (B) MeOH with 0.1% formic acid. The flow rate was set at 500 µL/min. The elution profile was 0–0.5 min, 75% B; 0.5–2 min, 75–100% B; 2–4 min, 100% B; 4–4.1 min, 75% B; 4.1–5.0, 75% B to equilibration to the initial conditions. The overall run time was 5 min, and the injection volume was 20.0 µL. The LC effluent was pumped to a Q-Exactive mass spectrometer (Q Exactive Plus-Ultimate 3000 HPLC system, Thermo Scientific, Bremen, Germany), operating in positive ionization mode. The spray voltage was set at 3.9 and 2.9 kV in positive ionization mode. The capillary temperature was 380 °C, and the S-lens radio frequency (RF) level was set at 80 (arbitrary units). The nitrogen sheath and auxiliary gas flow rates were set at 60 and 20 (arbitrary units). To ensure mass accuracies below 6 ppm, the instrument was calibrated in positive mode using the manufacturer’s calibration solution (Thermo Fisher Scientific, Bremen, Germany). The mass spectrometer acquired FullMS and T-SIM at a resolution of 70.000 full width at half maximum (FWHM) and with an automatic gain control (AGC) of 106, maximum IT 75 ms. The target masses were 348.2890 *m*/*z* to AEA, 379.2837 *m*/*z* to 2-AG, 352.3143 *m*/*z* to D4-AEA, and 384.3153 *m*/*z* to D5-2AG.

### 2.5. Statistical Analysis

The sample size of the present study was not calculated a priori because this was a probabilistic sampling cohort. However, a posteriori analysis was performed using the G*Power program v.3.1.9.6 [43] and the milk concentration of leptin, AEA, and 2-AG as parameters. Regarding leptin, one of the main hormonal hallmarks of obesity, the inclusion of at least 14 women per group would result in a statistical power of 80% (1-β = 0.8) to identify statistically significant differences (α = 0.05), with an effect size of d = 0.99, a two-tailed test, and a sample size ratio = 1. For the endocannabinoids, AEA and 2-AG, considering the same statistical power, a sample size of 38 and 24, respectively, would be used per group. Using the actual sample concentrations obtained in the present study, the effect size for leptin at T2 and pre-pregnancy BMI was d = 1.06, for AEA at T2 was d = 0.46, and for 2-AG at T2 was d = 0.56.

The distribution of the continuous variables was evaluated according to the Shapiro-Wilk test. Because those variables (milk compounds) did not present normal distribution, non-parametric tests were performed: 1-Mann–Whitney test was used to compare each compound concentration per time point separately (BMI < 25 vs. BMI ≥ 25 or n GWG vs. e GWG), and 2-Kruskal–Wallis test with Dunn’s post hoc test was used to evaluate the time variation of each compound in the breast milk, considering all the samples without categorization. The dietary intake data were adjusted according to the total energy consumed by the participants using the residues method [42].

Spearman correlations were performed between variables of maternal dietary intake during pregnancy and maternal anthropometric variables and the content of triglycerides, proteins, endocannabinoids, and hormones in milk at all time points. For this analysis, a moderate correlation was considered at 0.25 > R^2^ < 0.50, a high correlation at R^2^ > 0.50, and a low correlation at R^2^ < 0.25.

Linear regression models were used to investigate the association of anthropometric variables and lipid dietary intake and the composition of endocannabinoids in milk, considering a single sample set from 2 to 119 days with only one sample from each woman (n = 84). For this analysis, all women with milk samples at T2 (n = 60), women with samples at T1 (n = 15, including those with samples also at the last visit), and those with samples exclusively at T3 (n = 9) were included.

The confounders were selected according to a theoretical model based on the scientific literature. Additionally, the human milk compounds that showed significant correlations with endocannabinoids were used as adjustments. The following variables were used: maternal age, parity, years of schooling, postpartum time of the sample, and milk triglycerides and leptin. Statistical analyses were performed using Prisma software, version 9.0, and RStudio version 4.1.2 (R Core Team, 2021). A significance level < 0.05 was used for all analyses.

## 3. Results

### 3.1. Anthropometric and Demographic Profile of the Participants

In this study, 147 women were recruited and signed the term of consent form. Forty-nine women were excluded at the beginning of the study because of missing visits, changing the living area, or not meeting the inclusion criteria. Additionally, six participants did not collect milk samples. A total of 92 participants were included in this study, from whom 149 milk samples were collected: 41 samples in T1 (2–8 postpartum days), 67 samples in T2 (28–50 postpartum days), and 41 samples in T3 (88–119 postpartum days). Eight women were excluded from the FFQ analysis due to missing data or implausible energy intake (over 6000 kcal) (n = 84). Only six participants had samples collected across the three postpartum periods (Figure 1).

The median maternal age was 26 years old, and most of the participants had vaginal delivery (58.8%), and there was a higher prevalence of Black/Hispanic women in this cohort (60.9%). The median pre-pregnancy body mass index (BMI) and gestational weight gain (GWG) were 24.4 kg/m^2^ and 11.8 kg, respectively. Among the participants, 44 women were normal weight (51%), 26 were overweight (30%), and 14 were obese (16%). Two women had a BMI < 18.5 (18.2 and 18.3 kg/m^2^). The median energy intake per day was 2740 kcal (113 g protein, 432 g carbohydrates, and 84.6 g of total lipids). The *n*-6/*n*-3 ratio was 10.6 (Table 1).

### 3.2. Macronutrient and Hormone Profile in Human Milk

Milk protein concentration was higher at T1 (median: 16.26 mg/mL) compared with T2 (median: 13.49 mg/mL) (*p* = 0.0157) but not compared with T3 (median: 15.42 mg/mL). Milk triglyceride concentration was higher in T2 (median: 964.3) compared with T3 (median: 779.4 mg/mL) (*p* = 0.0426) but not compared with T1 (median: 842.4 mg/mL). Leptin and insulin milk concentration did not vary across the three-time points analyzed (Table 2).

Milk protein concentration was not altered in overweight or obese women or women with excessive GWG at any time point analyzed, but triglyceride concentration was 19.8% higher in milk samples from overweight or obese women at T3 compared with normal-weight women (*p* = 0.0195). Maternal pre-pregnancy overweight or excessive GWG did not account for insulin milk concentration variations. However, milk leptin concentration was 88.8% higher at T2 and 2-fold higher at T3 in overweight or obese women than in normal-weight women (*p* < 0.0001). In addition, leptin concentration was 63.6% higher in T2 milk samples from women presenting excessive GWG (*p* = 0.0141) (Table 3).

### 3.3. Endocannabinoid Profile in Human Milk

**Figure 2:**
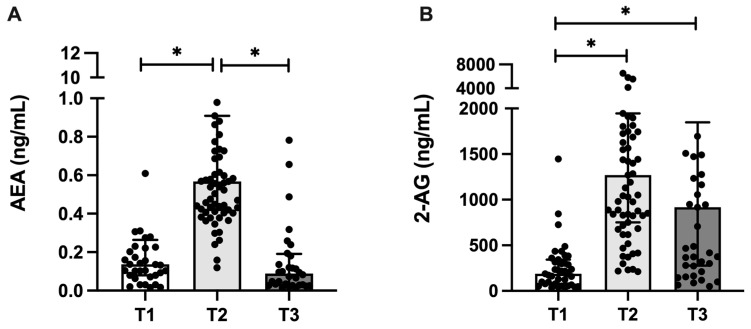
Endocannabinoid concentration in the human milk between 2 and 119 days postpartum. Milk concentration of the endocannabinoids (**A**) anandamide (AEA) and (**B**) 2-arachidonoylglycerol (2-AG) at 2–8 (T1, n = 41), 28–47 (T2, n = 67) and 88–119 (T3, n = 41) days postpartum. Data are expressed as median and interquartile intervals and were analyzed using Kruskal–Wallis test, followed by Dunn’s post-hoc test. * *p* < 0.05.

AEA and 2-AG milk concentration varied across lactation, with higher levels of AEA in T2 (median: 0.568 ng/mL), compared with T1 (median: 0.136 ng/mL) and T3 (median: 0.088 ng/mL) (*p* < 0.0001), and higher levels of 2-AG in T2 (median: 1269 ng/mL) and T3 (median: 916.6 ng/mL), compared with T1 (median: 187.8 ng/mL) (*p* < 0.001) (Figure 2). In addition, 2-AG milk concentration was approximately 1000-fold higher than the AEA concentration (Figure 2).

**Figure 3:**
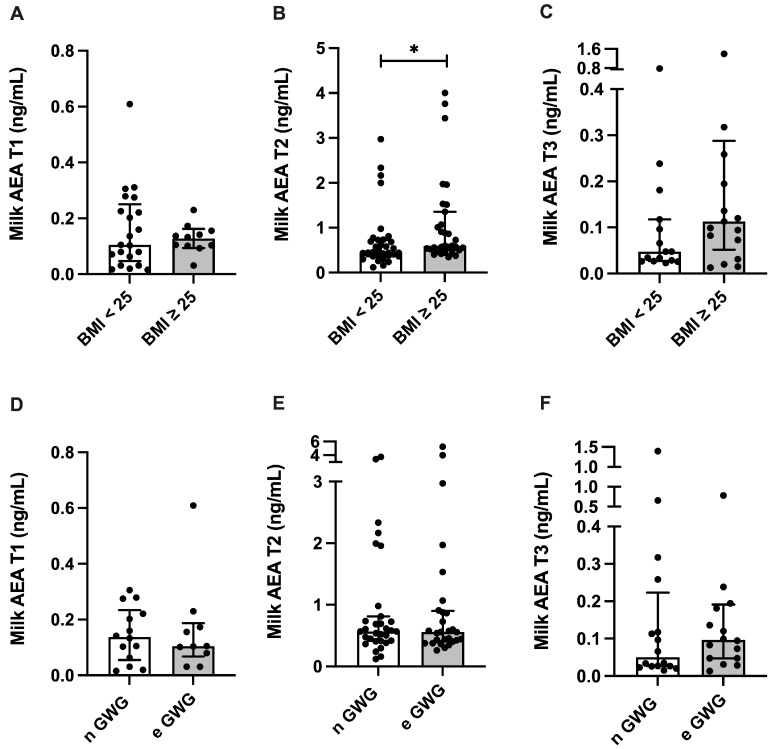
Milk concentration of the endocannabinoid anandamide (AEA) and maternal anthropometry. (**A**–**C**) AEA milk concentration at 2–8 (T1, n = 41), 28–47 (T2, n = 67), and 88–119 (T3, n = 41) days postpartum stratified by maternal pre-pregnancy body mass index (BMI; <25 or >25) or (**D**–**F**) gestational weight gain (GWG; normal (n GWG) or excessive (e GWG)). Data are expressed as median and interquartile intervals and were analyzed using Mann–Whitney test. * *p* < 0.05.

**Figure 4:**
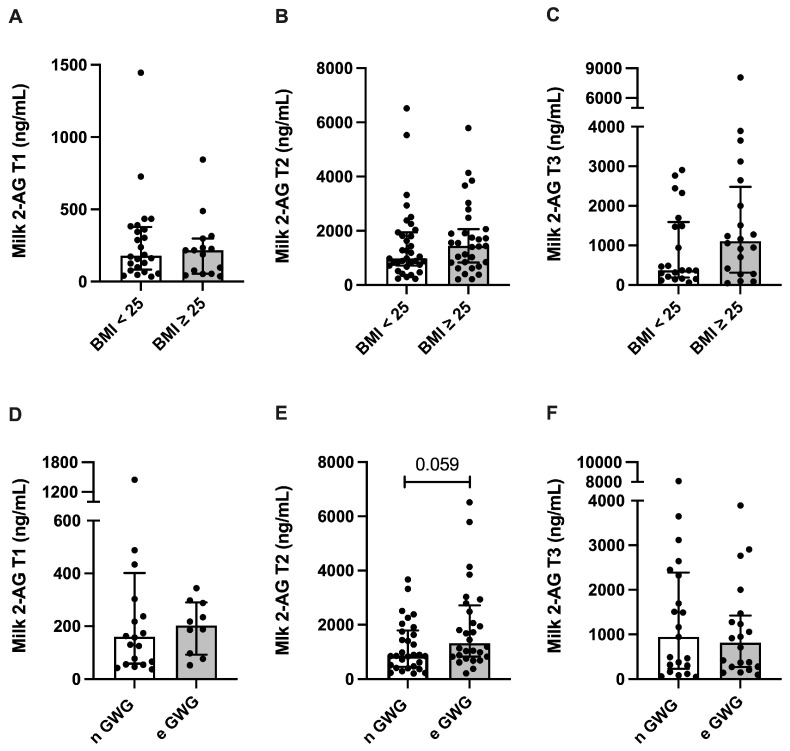
Milk concentration of the endocannabinoid 2-arachidonoylglycerol (2-AG) and maternal anthropometry. (**A**–**C**) 2-AG milk concentration at 2–8 (T1, n = 41), 28–47 (T2, n = 67), and 88–119 (T3, n = 41) days postpartum stratified by maternal pre-pregnancy body mass index (BMI; <25 or >25) or (**D**–**F**) gestational weight gain (GWG; normal (n GWG) or excessive (e GWG)). Data are expressed as median and interquartile intervals and were analyzed using Mann–Whitney test.

AEA milk concentration was 32.9% higher in overweight or obese women at T2, compared with normal-weight women (*p* = 0.0154), while excessive GWG did not alter AEA milk levels (Figure 3). The 2-AG milk concentration was not altered in overweight or obese women, but the 2-AG milk concentration in women presenting excessive GWG was 51.6% higher at T2 compared with normal GWG (*p* = 0.059) (Figure 4).

### 3.4. Spearman Correlations

**Figure 5:**
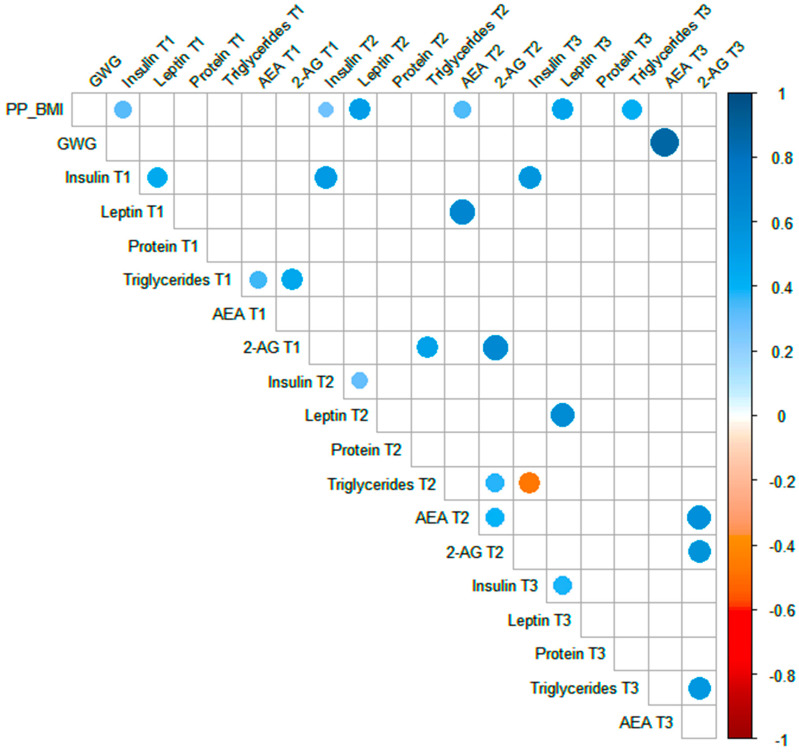
Spearman rank correlations between milk components and maternal anthropometry. Spearman correlations between milk concentration of insulin, leptin, proteins, triglycerides, and endocannabinoids (AEA and 2-AG) at postpartum days 2–8 (T1), 28–47 (T2), and 88–119 (T3) and maternal pre-pregnancy BMI (PP_BMI) and gestational weight gain (GWG). The circle only becomes visible for the correlations with a significance level of *p* < 0.05. The circles indicate statistical significance (*p* < 0.05), and their size is directly proportional to the effect size. The blue color indicates a positive correlation, and the red color indicates a negative correlation, according to the scale bar.

**Figure 6:**
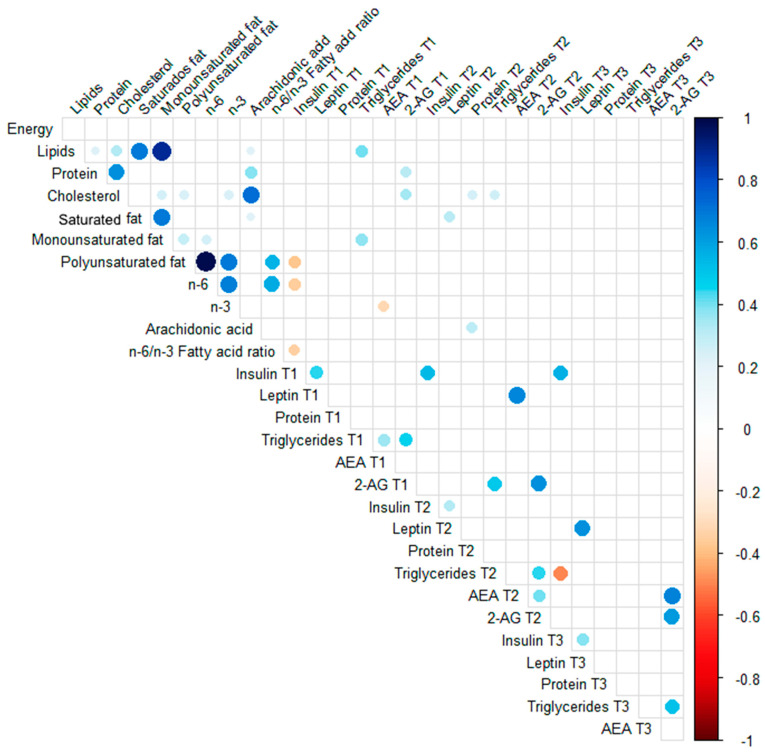
Spearman rank correlations between milk components and maternal diet. Spearman correlations between milk concentration of insulin, leptin, proteins, triglycerides, and endocannabinoids (AEA and 2-AG) at postpartum days 2–8 (T1), 28–47 (T2), and 88–119 (T3) and maternal total energy intake and the consumption of dietary lipids, proteins, cholesterol, saturated, monounsaturated, and polyunsaturated fat, *n*-3 PUFA, *n*-6 PUFA, arachidonic acid, and *n*-6/*n*-3 ratio. The circles indicate statistical significance (*p* < 0.05), and their size is directly proportional to the effect size. The blue color indicates a positive correlation, and the red color indicates a negative correlation, according to the scale bar.

Maternal anthropometric characteristics were significantly correlated with several milk components across lactation (Figure 5). Of note, maternal pre-pregnancy BMI was directly correlated with milk concentration of insulin (T1 and T2), leptin (T2 and T3), AEA (T2), and triglycerides (T3) (*p* < 0.05; 0.25 > R^2^ < 0.50). The maternal GWG was strongly and directly correlated with AEA milk concentration at T3 (*p* < 0.05; R^2^: 0.87). It was also observed a direct correlation between milk triglycerides and 2-AG concentration at T3 (*p* < 0.05; R^2^: 0.47) (Figure 5). The evaluation of correlations between maternal lipid intake and milk components was less prominent (Figure 6). Maternal intake of *n*-6 PUFA was inversely correlated with milk insulin concentration at T1 (*p* < 0.05; R^2^: −0.35), and the *n*-3 PUFA intake was inversely correlated with milk AEA concentration at T1 (*p* < 0.05; R^2^: −0.31).

### 3.5. Linear Regression Models

The unadjusted linear regression models showed a positive association between excessive GWG and the concentration of 2-AG in breast milk. Thus, women with excessive GWG had a 1366 ng/mL higher concentration of 2-AG in breast milk than those without excessive GWG (β = 1366; 95%CI: 362.4; 2370.7). After adjustments for triglyceride and leptin concentration in breast milk, maternal age, parity, and the analyzed lactation period, the positive linear association remained significant and was even stronger among women with excessive GWG and 2-AG content in milk (β = 1629.5; 95%CI: 466.7; 2792.3), compared with women without excessive GWG (Table 4). No significant associations were found between maternal dietary intake and the levels of endocannabinoids in breast milk (Table 5).

## 4. Discussion

In the present study, the main finding was that maternal pre-pregnancy overweight or obesity and excessive gestational weight gain were associated with increased levels of the endocannabinoids AEA and 2-AG in mature breast milk but not in colostrum. A significant variation in endocannabinoid concentrations was observed from colostrum to mature milk, with mature milk exhibiting higher concentrations. Notably, milk endocannabinoids were positively correlated with milk triglyceride and leptin levels and negatively correlated with maternal intake of *n*-3 PUFA during the third trimester of gestation.

Endocannabinoids are lipid-derived molecules, and a positive association between their levels and the lipid content of breast milk is expected. The median triglyceride concentration in breast milk was significantly higher at one month postpartum (T2) compared with three months (T3), which occurred parallel to an increase in the endocannabinoids AEA and 2-AG. Differently, the protein concentration was higher in colostrum (T1) when lower levels of endocannabinoids were exhibited. Higher protein concentration in colostrum has been observed in previous studies [44], and it has been associated with the content of proteins related to energy functions, the immune system, and bioactive compounds such as peptide-derived hormones and growth factors [45,46].

Milk samples of women with pre-pregnancy overweight or obesity showed higher triglyceride concentration compared with milk from women of normal weight at T3, with a positive correlation between these variables at the same time point. The association between maternal BMI and milk macronutrients varies across studies [47,48], likely due to differences in the source of lipids in breast milk, which may come from the diet or maternal energy stores, such as adipose tissue [49]. However, we found no marked correlations between milk components and maternal diet, possibly because the FFQ was applied to the volunteers during the third gestational trimester, while milk samples were collected up to three months postpartum. Thus, we speculate that the higher triglyceride content in breast milk of overweight or obese women is associated with increased mobilization from adipose tissue stores.

The adipocyte-derived hormone leptin and the pancreas-derived hormone insulin are also present in breast milk. These hormones are important for energy metabolism by inducing satiety and energy expenditure, but they also promote early development of the hypothalamic feeding circuitry [50,51,52]. In the present study, a robust increase in leptin concentration was observed in the milk of overweight or obese women compared with the women of normal weight. A similar profile was observed in the mature milk of women presenting excessive GWG. Spearman correlation analysis showed that leptin concentration in mature milk was positively correlated with maternal pre-pregnancy BMI, while insulin concentration in colostrum was positively correlated with maternal BMI. These findings corroborate other human studies on milk hormone composition [53,54,55,56,57]. Altered leptin levels in early life may increase the risk of overweight and obesity in offspring later in life [50,58]. Specifically, milk leptin concentration at one month postpartum has been positively associated with infant BMI up to two years of age [54].

Obesity is frequently associated with increased circulating levels of endocannabinoids [24,59,60,61], but the effect of maternal obesity on the endocannabinoid milk profile is poorly investigated. In the present study, maternal obesity or overweight and excessive GWG were associated with higher concentrations of endocannabinoids in mature milk, confirming the main hypothesis of this study. In contrast, a cohort study conducted in the United States did not find differences in milk 2-AG concentration between normal weight and overweight or obese women at four to eight weeks postpartum, and milk AEA concentration was reported to be very low and under the limit of detection [62]. This divergence between the studies may be attributed to (1) differences in sample size, as Datta et al. (2021) [62] included only 11–13 samples per group, and we analyzed 31–35 samples per group at a comparable time point (T2), and (2) differences in the timing of sample collection (morning vs. 24 h collection). Interestingly, Datta et al. reported higher levels of milk 2-AG during daytime compared with nighttime [62]. This physiological circadian rhythm has also been demonstrated for the serum levels of 2-AG [63]. This rhythm may contribute to feeding behavior during the day and decreased hypothalamus-pituitary-adrenal (cortisol) activity at night, modulated by the ECS [25]. More recently, Fradet et al. (2024) demonstrated that milk 2-AG concentration at two months postpartum is higher in samples of overweight or obese women with gestational diabetes compared with obese women who did not develop this comorbidity [35]. In addition, they found a negative correlation between non-*n*-3 N-acyl-ethanolamines (a subset of endocannabinoids) and the weight-for-age z-score of offspring from women with gestational diabetes [35]. Therefore, we suggest that both obesity and gestational diabetes may increase the levels of endocannabinoids in breast milk, potentially impacting offspring development.

In the present study, we observed that the concentration of 2-AG is 1000-fold higher than AEA across the three lactation time points, consistent with relative concentrations observed in other biological samples, such as the brain [64,65], and with previous studies reporting the absolute concentrations of these endocannabinoids in human milk [32,33,34,62,66,67]. To the best of our knowledge, this is the first study to evaluate endocannabinoid levels from colostrum to mature milk in both eutrophic and overweight or obese women. We observed that milk AEA peaked around one month postpartum (T2), and 2-AG levels also increased at T2 but remained in high concentrations for up to three months (T3). This profile is not yet fully understood, but it may be associated with neurodevelopmental processes occurring primarily after birth, such as synaptogenesis, myelinization, and synaptic pruning [68], as well as the initiation of suckling [69]. Corroborating the present results, it was demonstrated that the concentration of the endocannabinoid-like compound palmitoylethanolamide is increased in breast milk at six months compared with one month in a cohort of Guatemalan women [33]. In contrast, no differences in endocannabinoid concentration were observed between transitional milk at two weeks and mature milk at four weeks postpartum [32], or between mature milk at four to eight weeks and mature milk at four to six months postpartum [62] in two U.S. cohorts.

The main limitations of this study include (1) the incomplete emptying of the mammary glands during milk extraction, which may affect the lipid content of breast milk; (2) the lack of milk fatty acid data, which would be relevant given that endocannabinoids are derived from arachidonic acid (*n*-6 PUFA); and (3) the follow-up losses resulting in only six women with milk collected in all time points of lactation. This study was performed in a non-probabilistic sample, which limits the generalization of the findings. Although using longitudinal regression models with repeated measures (the ideal statistical approach for this study design) was not possible, using statistical models that accounted for adjustments of potential confounders was one strategy to ensure that the findings could be considered reliable. The strengths of the present study include (1) the evaluation of milk endocannabinoids at important time points of lactation (T1, T2, T3) according to maternal BMI, GWG, and dietary profile and (2) it is the first study conducted in a Brazilian population, using a greater sample size compared with previous studies on milk endocannabinoids.

## 5. Conclusions

The milk levels of the endocannabinoids AEA and 2-AG change differentially across breastfeeding time points, with a transient increase in AEA at one month postpartum and a sustained increase in 2-AG from one to three months. In addition, there was a negative association between maternal intake of *n*-3 PUFA and the milk levels of endocannabinoids. Maternal overweight or obesity and excessive GWG were associated with increased levels of both endocannabinoids, but there was stronger evidence of a positive association between excessive GWG and the milk levels of 2-AG.

The precise physiological relevance of the amounts of endocannabinoids in breast milk for offspring development remains to be determined, possibly using experimental models of maternal obesity as a more controlled approach. Future studies should also explore the association between elevated endocannabinoid levels in the breast milk of obese women and infant development. Assessing endocannabinoid levels in breast milk may have clinical implications for evaluating the risk of metabolic and neurodevelopmental complications in offspring. On the other hand, higher endocannabinoid levels in milk might benefit babies born with low birth weight, as these compounds could support positive energy balance and adequate brain development. In addition, nutritional interventions targeting maternal diet during breastfeeding, such as modulating dietary *n*-3 and *n*-6 PUFA, may be an interesting strategy for altering the levels of endocannabinoids in breast milk.

## Figures and Tables

**Table 1:** Demographic, reproductive, anthropometric, and dietetic profile of women from the Brazilian cohort, Rio de Janeiro.

Variables	n = 92
	Median [IQR]
Maternal age (years)	26.0 [22.2–31.4]
Education (schooling years)	12.0 [9.00–12.0]
Pre-pregnancy BMI (kg/m^2^)	24.4 [21.1–28.8]
Total GWG (kg)	11.8 [9.00–14.3]
Dietary intake	
Total energy intake (kcal)	2740 [1950–3800]
Proteins (g) ^1^	113 [100–128]
Carbohydrates (g) ^1^	432 [389–461]
Lipids (g) ^1^	84.6 [75.1–93.3]
Saturated fatty acid (g) ^1^	34.1 [29.6–39.0]
Monounsaturated fatty acid (g) ^1^	28.5 [24.9–31.1]
Polyunsaturated fatty acid (g) ^1^	18.2 [15.6–22.0]
Omega-6 polyunsaturated fatty acid (g) ^1^	16.2 [14.1–19.7]
Omega-3 polyunsaturated fatty acid (g) ^1^	1.49 [1.28–1.72]
Arachidonic acid (g) ^1^	0.0936 [0.0702–0.134]
*n*-6/*n*-3 ratio	10.6 [9.60–13.0]
Gestational age at delivery	39.4 [38.9–40.9]
Delivery mode ^2^	**n (%)**
Vaginal	50 (58.8)
Caesarean	35 (41.2)
Parity ^3^	
Multiparous	42 (46.2)
Primiparous	49 (53.8)
Skin color ^4^	
White	11 (12.2)
Brown/Mixed color	56 (62.2)
Black	21 (23.3)
Yellow/Asian	2 (2.2)
Pregestational BMI (kg/m^2^) ^5^	
Underweight (<18.5)	2 (2.3)
Normal weight (≥18.5 and <25.0)	44 (50.6)
Overweight (≥25.0 and <30.0)	26 (29.9)
Obese (≥30.0)	14 (16.1)

Note: ^1^ Data corrected by total kcal of maternal intake. IQR: interquartile range; BMI: body mass index; GWG: gestational weight gain. ^2^ Because of missing data n = 85. ^3^ Because of missing data n = 91. ^4^ Because of missing data n = 90. ^5^ Because of missing data n = 86.

**Table 2:** Milk concentration of total proteins, triglycerides, insulin, and leptin across lactation of women from the Brazilian cohort, Rio de Janeiro.

Variables	Median [IQR] (n)		
	T1	T2	T3
Total protein (mg/mL)	16.3 [15.0–18.1] * (40)	13.5 [12.5–15.5] (65)	15.4 [14.6–17.5] (39)
Total triglycerides (mg/mL)	842.4 [773–966] (41)	964.3 [848–1074] ^#^ (64)	779.4 [674–903] (41)
Insulin (ng/mL)	0.90 [0.55–1.21] (41)	0.84 [0.70–1.21] (67)	1.04 [0.87–1.70] (37)
Leptin (ng/mL)	0.45 [0.34–0.72] (31)	0.65 [0.45–0.75] (67)	0.62 [0.44–0.81] (41)

Data were analyzed using Kruskal–Wallis test, followed by Dunn’s multiple comparison test. * *p* < 0.05 compared with T2; ^#^ *p* < 0.05 compared with T3.

**Table 3:** Milk concentration of total proteins, triglycerides, insulin, and leptin across lactation of Brazilian women according to pregestational BMI or gestational weight gain (GWG).

Variables	Median [IQR] (n)	
	BMI < 25	BMI ≥ 25
Total protein (mg/mL) T1	16.0 [14.3–18.1] (24)	17.3 [14.5–19.8] (16)
Total protein (mg/mL) T2	12.6 [11.7–15.3] (35)	15.2 [13.0–17.1] (30)
Total protein (mg/mL) T3	15.1 [13.8–19.1] (20)	15.5 [13.6–17.3] (19)
Total triglycerides (mg/mL) T1	912 [786–1113] (25)	823 [704–1036] (16)
Total triglycerides (mg/mL) T2	1036 [803–1126] (35)	855 [788–1071] (29)
Total triglycerides (mg/mL) T3	693 [605–851] (21)	830 [767–1008] * (20)
Insulin (ng/mL) T1	0.58 [0.44–1.17] (25)	1.14 [0.85–1.49] (16)
Insulin (ng/mL) T2	0.75 [0.61–1.21] (35)	1.08 [0.71–1.56] (32)
Insulin (ng/mL) T3	1.04 [0.79–2.02] (21)	1.23 [0.88–1.75] (20)
Leptin (ng/mL) T1	0.45 [0.16–0.77] (22)	0.46 [0.35–0.89] (16)
Leptin (ng/mL) T2	0.45 [0.32–0.65] (35)	0.85 [0.63–1.15] * (32)
Leptin (ng/mL) T3	0.44 [0.25–0.63] (21)	0.95 [0.62–1.27] * (20)
	**n GWG**	**e GWG**
Total protein (mg/mL) T1	16.4 [14.8–18.4] (28)	15.6 [14.5–19.8] (12)
Total protein (mg/mL) T2	12.6 [11.6–15.0] (36)	15.9 [13.3–18.1] (28)
Total protein (ng/mL) T3	15.9 [13.7–19.3] (21)	15.1 [14.4–17.3] (20)
Total triglycerides (ng/mL) T1	832 [742–1036] (29)	844 [704–1139] (12)
Total triglycerides (mg/mL) T2	1014 [803–1084] (36)	951 [796–1084] (28)
Total triglycerides (mg/mL) T3	779 [605–1008] (21)	798 [666–967] (19)
Insulin (ng/mL) T1	0.73 [0.44–1.49] (21)	1.01 [0.58–1.22] (20)
Insulin (ng/mL) T2	0.96 [0.61–1.51] (31)	0.78 [0.68–1.29] (36)
Insulin (ng/mL) T3	1.44 [0.81–3.12] (15)	1.02 [0.87–1.70] (26)
Leptin (ng/mL) T1	0.50 [0.24–0.67] (18)	0.40 [0.34–0.93] (20)
Leptin (ng/mL) T2	0.44 [0.32–0.75] (31)	0.72 [0.59–1.03] * (36)
Leptin (ng/mL) T3	0.62 [0.24–1.16] (15)	0.62 [0.38–0.97] (26)

Data were analyzed using Mann–Whitney test. * *p* < 0.05 compared with BMI < 25 or non-excessive gestational weight gain (n GWG).

**Table 4:** Linear regression models of associations between pre-gestational BMI and gestational weight gain and AEA and 2-AG concentrations in breast milk at 2–119 days postpartum (n = 84).

Independent Variables		Unadjusted models	
		AEA	
**Categorical Variable (yes/no)**	**β**	**CI (95%)**	***p***
BMI ≥ 25 kg/m^2^	0.64	–0.97; 2.26	0.432
Gestational Weight Gain ^1^	2.37	–1.65; 6.38	0.239
	**2-AG**		
**Categorical Variable (yes/no)**	**β**	**CI (95%)**	***p***
BMI ≥ 25 kg/m^2^	174.60	–398.5; 747.6	0.546
Gestational Weight Gain^1^	1366.6	362.4; 2370.7	**0.009**
**Independent Variables**		**Adjusted models**	
		**AEA**	
**Categorical Variable (yes/no)**	**β**	**CI (95%)**	***p***
BMI ≥ 25 kg/m^2^	1.81	–0.18; 3.80	0.073
Gestational Weight Gain ^1^	3.51	–1.22; 8.23	0.140
		**2-AG**	
**Categorical Variable (yes/no)**	**β**	**CI (95%)**	***p***
BMI ≥ 25 kg/m^2^	413.93	–251.9; 1079.8	0.219
Gestational Weight Gain ^1^	1629.51	466.7; 2792.3	**0.008**

Note: ^1^ Classification of gestational weight gain according to the Institute of Medicine (IOM, 2009). Adjustments for maternal age, parity, postpartum time of the sample, milk triglycerides, and leptin. Bold *p*-values indicate a statistical significance. CI: confidence interval; AEA: anandamide; 2-AG: 2-arachidonoylglycerol.

**Table 5:** Linear regression models of associations between dietary lipid intake during pregnancy and AEA and 2-AG concentration in breast milk from 2–119 days postpartum (n = 84).

Independent Variables	Unadjusted Models		
		AEA	
Dietary intake	β	CI (95%)	*p*
Total lipids (g)	−0.01	−0.05; 0.04	0.807
Cholesterol (mg)	−0.00	−0.00; 0.00	0.608
Saturated fatty acid (g)	−0.03	−0.11; 0.05	0.429
Monounsaturated fatty acid (g)	−0.01	−0.15; 0.14	0.909
Polyunsaturated fatty acid (g)	−0.02	−0.14; 0.10	0.756
Fatty acid *n*-6 (g)	−0.02	−0.15; 0.11	0.785
Fatty acid *n*-3 (g)	−0.33	−2.31; 1.66	0.745
Arachidonic acid (g)	5.24	−5.54; 16.03	0.336
Ratio *n*-6/*n*-3	−0.01	−0.25; 0.24	0.952
	2-AG		
Dietary intake	β	CI (95%)	*p*
Total lipids (g)	−5.33	−21.09; 10.43	0.503
Cholesterol (mg)	−0.19	−1.50; 1.11	0.770
Saturated fatty acid (g)	−14.81	−42.38; 12.77	0.382
Monounsaturated fatty acid (g)	−22.68	−74.03; 28.66	0.378
Polyunsaturated fatty acid (g)	8.00	−35.59; 51.61	0.716
Fatty acid *n*-6 (g)	13.52	−32.12; 59.17	0.557
Fatty acid *n*-3 (g)	114.6	−598.50; 827.77	0.745
Arachidonic acid (g)	1492.3	−2393.96; 5378.56	0.447
Ratio *n*-6/*n*-3	60.38	−26.32–147.08	0.170
		AEA	
Dietary intake	β	CI (95%)	*p*
Total lipids (g)	0.01	−0.05; 7.41	0.815
Cholesterol (mg)	0.00	−0.00; 0.00	0.716
Saturated fatty acid (g)	−0.03	−0.11; 0.05	0.416
Monounsaturated fatty acid (g)	−0.01	−0.16; 0.13	0.850
Polyunsaturated fatty acid (g)	−0.01	−0.14; 0.12	0.886
Fatty acid *n*-6 (g)	−0.01	−0.15; 0.13	0.861
Fatty acid *n*-3 (g)	0.01	−2.02; 2.18	0.944
Arachidonic acid (g)	4.37	−7.00; 15.75	0.446
Ratio *n*-6/*n*-3	−0.01	−0.29; 0.24	0.847
	2-AG		
Dietary intake	β	CI (95%)	*p*
Total lipids (g)	−2.98	−18.13; 12.17	0.696
Cholesterol (mg)	−0.38	−1.65; 0.89	0.557
Saturated fatty acid (g)	−8.73	−36.13; 18.67	0.528
Monounsaturated fatty acid (g)	−22.63	−71.68; 26.41	0.361
Polyunsaturated fatty acid (g)	−12.63	−56.26; 30.98	0.566
Fatty acid *n*-6 (g)	−10.59	−56.76; 35.57	6.649
Fatty acid *n*-3 (g)	−50.32	−756.26; 655.63	0.887
Arachidonic acid (g)	2039.36	−1766.42; 5845.14	0.290
Ratio *n*-6/*n*-3	22.26	−66.14; 110.66	0.617

Note: Adjustments for maternal age, parity, postpartum time of the sample, triglycerides, and leptin. CI: confidence interval; AEA: anandamide; 2-AG: 2-arachidonoylglycerol.

## Data Availability

The original contributions presented in this study are included in the article. Further inquiries can be directed to the corresponding author.
